# Transcription factors regulate GPR91-mediated expression of VEGF in hypoxia-induced retinopathy

**DOI:** 10.1038/srep45807

**Published:** 2017-04-04

**Authors:** Tingting Li, Jianyan Hu, Fengjuan Gao, Xinhua Du, Yongdong Chen, Qiang Wu

**Affiliations:** 1 Department of Ophthalmology, the Sixth People’s Hospital, Shanghai Jiaotong University, 600 Yishan Road, Shanghai 200233, China; 2 Shanghai Key Laboratory of Diabetes Mellitus, Shanghai 200233, China

## Abstract

Hypoxia is the most important factor in the pathogenesis of diabetic retinopathy (DR). Our previous studies demonstrated that G protein-coupled receptor 91(GPR91) participated in the regulation of vascular endothelial growth factor (VEGF) secretion in DR. The present study induced OIR model in newborn rats using exposure to alternating 24-hour episodes of 50% and 12% oxygen for 14 days. Treatment with GPR91 shRNA attenuated the retinal avascular area, abnormal neovascularization and pericyte loss. Western blot and qRT-PCR demonstrated that CoCl_2_ exposure promoted VEGF expression and secretion, activated the ERK1/2 signaling pathways and upregulated C/EBP and AP-1. Knockdown of GPR91 inhibited ERK1/2 activity. GPR91 siRNA transduction and the ERK1/2 inhibitor U0126 inhibited the increases in C/EBP β, C/EBP δ, c-Fos and HIF-1α. Luciferase reporter assays and a chromatin immunoprecipitation (ChIP) assay demonstrated that C/EBP β and c-Fos bound the functional transcriptional factor binding site in the region of the VEGF promoter, but not C/EBP δ. Knockdown of C/EBP β and c-Fos using RNAi reduced VEGF expression. Our data suggest that activation of the GPR91-ERK1/2-C/EBP β (c-Fos, HIF-1α) signaling pathway plays a tonic role in regulating VEGF transcription in rat retinal ganglion cells.

Vascular endothelial growth factor (VEGF) is a prominent angiogenic factor that drives the proliferation and migration of vascular endothelial cells and induces vascular permeability^1^,^2^. Hypoxia is an important stimulus that leads to VEGF upregulation in various retinopathies. The retina is the most metabolically active human tissue, and it is highly sensitive to hypoxia^3^. Many retinal diseases are related to hypoxia, including diabetic retinopathy (DR), retinopathy of prematurity (ROP) and occlusive diseases of retinal vessels. Sapieha *et al*.^4^ presented a strong correlation between G protein-coupled receptor 91(GPR91) and VEGF in hypoxia-induced retinopathy.

G protein-coupled receptors (GPCRs) constitute the largest family of cell surface protein receptors, and GPCRs are involved in a variety of physiological and pathological processes^5^. GPR91 is activated by specific binding with extracellular succinate, and it is associated with neovascularization^4^,^6^. Our previous research showed that an accumulation of succinate-mediated GPR91 activation promoted VEGF release and increased retinal vascular disorders in the early stage of DR^7^,^8^. Additionally, we also observed that the GPR91-regulated overexpression of VEGF was achieved via activation of the ERK1/2/COX-2/ PGE_2_ signaling pathway *in vivo* and *in vitro*^7^,^8^,^9^. ERK1/2 signaling is a significant signal transfer hub that transmits the upstream signal to the cytoplasm protein and transfers extracellular stimuli inside the cell and to the nucleus, which eventually facilitates transcription factor expression in the nuclei and exerts a regulatory function^10^,^11^. Transcription factors combine with gene regulation sequences and participate in the process and regulation of gene transcription. Understanding the mechanisms of VEGF transcriptional regulation is of paramount importance to expand our knowledge of GPR91-dependent VEGF expression and enhance the sensitivity and specificity of genome-wide efforts to characterize it.

Our preliminary work, used an online transcription factor database (http://www.cbrc.jp/research/db/TFSEARCH.html) to analyze the promoter sequence upstream of the VEGF-A (simply VEGF hereafter) gene and identify putative binding sites for transcription factors. The predicted results with a score >90 (out of 100) and belonging to the rat source yielded two transcription factors: CCAAT enhancer binding protein (C/EBP) and activator protein-1 (AP-1), with scores of 93.1 and 90.7, respectively.

C/EBPs are a family of transcription factors, and six members have been isolated and characterized to date: C/EBP α, C/EBP β, C/EBP γ, C/EBP δ, C/EBP ε and C/EBP ζ. All C/EBP isoforms contain a highly conserved basic leucine zipper domain at the C-terminus that is involved in dimerization and DNA binding^12^. The C/EBPs play pivotal roles in numerous physiological and pathological responses, such as cellular proliferation, differentiation, immunoreaction, inflammatory processes, and tumor invasiveness and progression^13^,^14^. The expression of C/EBPs is regulated at multiple levels through the action of numerous factors, including hormones, mitogens, cytokines, nutrients and certain toxins^12^. Recent studies demonstrated roles for C/EBP β and C/EBP δ in the regulation of VEGF-C expression^15^,^16^. Extracellular regulated protein kinases 1/2 (ERK1/2) is the upstream signal that enhances the binding of C/EBPβ to the promoter region of VEGF-C^16^.

AP-1 also belongs to a family of basic leucine zipper transcription factors. It is a homo- and heterodimeric protein composed of proteins in the Jun, Fos, ATF and JDP subfamilies^17^. Each of these proteins is differentially expressed and regulated, which means that every cell type has a complex mixture of AP-1 dimers with subtly different functions^17^. AP-1 controls numerous cellular processes, including differentiation, proliferation and apoptosis^18^. Liu LZ *et al*.^19^ found that ERK1/2 signaling affected AP-1 and VEGF activity and induced angiogenesis. Ye *et al*.^13^,^20^ reported that AP-1 played a role in the regulation of ERK1/2-mediated VEGF secretion in Müller cells.

The present study, used the luciferase assay and chromatin immunoprecipitation (ChIP) assay to investigate a potential VEGF transcription factor and identified the transcription regulation mechanism of GPR91- dependent VEGF expression in hypoxic conditions. Our results support the potential therapeutic targets of hypoxic retinopathy.

## Methods

### Rat model of oxygen-induced retinopathy (OIR)

Litters of Sprague-Dawley rat pups and their mothers (SIPPR/BK Lab Animal Ltd, Shanghai, China) were transferred to oxygen exposure chambers within 8 hours after birth, and oxygen was cycled between 50% and 12% every 24 hours for 14 days. Age-matched control rats were maintained in room air (RA). The oxygen-exposed rats were returned to room air for up to 4 days after variable oxygen treatment to postnatal day (P)14 to allow time for retinal NV to develop. All animal procedures used in this study conformed to the Guide for Care and Use of Laboratory Animals published by the National Institutes of Health (Guide for the Care and Use of Laboratory Animals, 1996), and the Animal Ethics Committee of the Sixth People’s Hospital, Shanghai Jiaotong University approved the protocols.

The recombinant GPR91 shRNA (AACCCTAAATACAGTCTCATT), recombinant C/EBP β shRNA(ACAAGCTGAGCGACGAGTACA), recombinant c-Fos shRNA(GCCGGTCAAGAACATTAGCAA), and scrambled shRNA were designed and packaged by Genechem Co., Ltd (Shanghai, China), as previously described^7^. One microliter of GPR91 shRNA, C/EBP β shRNA, or c-Fos shRNA (1 μl of 1 × 10^8^ TU/ml) was delivered into the intravitreal space using a 30-gauge needle attached to a syringe (Hamilton Company, Reno, NY, USA) at the beginning of P8. Pups received an intravitreal injection of 0.1 mM U0126 (ERK1/2 inhibitor, Calbiochem, Gibbstown, NJ, USA) at P14, when retinal VEGF expression is high in this model.

### Retinal fluorescein–dextran perfusion

Pups were injected with a single intraperitoneal dose of 40 mg/kg sodium pentobarbital solution for anesthesia after P14, and a median sternotomy was performed. The left ventricle was perfused with 50 mg/ml of fluorescein isothiocyanate-dextran (2 × 10^6^ molecular weight, Sigma Chemical Co., St. Louis, MO) in PBS. The eyes were enucleated and fixed in 4% paraformaldehyde for at least 2 h in room air before removal of the retina. The peripheral retina was cut in 4 places and mounted using glycerol-gelatin. To determine retinal vascular development, computer-digitized images of fluorescein–dextran-stained retinas were observed and photographed under a fluorescence microscope (Olympus SZX16), and the fluorescence intensity of each image was analyzed using commercial software (Image Pro-Plus Media; Cybernetics, Silver Spring, MD). Images are presented at 2× magnification.

### Retinal ADPase staining

Pup retinas were dissected on day P18 and the retinal vasculature was stained for adenosine diphosphatase (ADPase) activity according to a standard protocol^21^. Digital images of all flattened, stained retinas were captured at 2× magnification to assess neovascularization. Images were saved using SPOT software (Diagnostic Instruments, Inc., Sterling Heights, MI, USA), an Olympus BH-2 microscope (McBain Instruments, Chatsworth, CA, USA), and a Dell Optiplex GX280 computer (Dell Computer Corporation, Dallas, TX, USA). The local retinal images of the ADPase-stained retinas containing pre-retinal vessel tufts are presented at 5× magnification.

### Retinal digest procedures

Enucleated eyes were fixed with 4% paraformaldehyde for 24 h. The retinas were rinsed in distilled water overnight and incubated with 3% trypsin in 0.1 M Tris buffer (pH 7.8) at 37 °C for 2 h. Non-vascular tissues were removed by gentle washing, and the isolated vasculature was flattened on polylysine-coated glass slides in distilled water for drying. Retinal blood vessels were subjected to periodic acid–Schiff (PAS) and hematoxylin staining. The capillary network was evaluated to identify the number of acellular capillaries using the quantitative methods of Huang H *et al*.^22^. The numbers of endothelial cells and pericytes were determined by counting respective nuclei under a microscope at 400× magnification. The six peripheral zone fields of retinal digest were analyzed for each retina in a masked fashion. The average number of endothelial cells and pericytes per mm^2^ vascular bed were calculated. The ratio of endothelial cells to pericytes was calculated.

### Primary retinal ganglion cell culture

Rat retinal cells were obtained and cultured as previously described^23^,^24^. Briefly, eyes from 1- to 4-day-old Sprague-Dawley rats were quickly enucleated, and the retinas were dissected and incubated in a 5 mg/ml papain dissociation solution (Worthington, Lakewood, NJ, USA) at 37 °C for 30 min. Retinal tissue was triturated by the addition of fresh ovomucoid solution (Sigma Aldrich, St. Louis, MO, USA), and the cell suspension was collected. The resulting retinal cell suspension was centrifuged at 400 × *g* for 10 min to separate retinal cells from the ovomucoid solution. The supernatant was discarded, and the remaining cells were resuspended in 10 ml of MEM containing 0.5 mg/ml BSA. Macrophages and microglial cells were removed by incubation of the cell suspension on a panning plate coated with an anti-rat-macrophage antiserum (1:50 in MEM) for 1 h at room temperature. Non-adherent cells were incubated for 1 h at 37 °C on a second panning plate coated with donkey anti-rabbit IgG (H + L) antibody (1:50, ProteinTech Group, Chicago, IL) and purified rat anti-mouse Thy 1.1 antibody (1:50, abcam, USA) for 1 h at 37 °C. The panning dish was washed 3 times with DPBS. Adherent cells were released from the plate by incubation with 0.125% trypsin for 5 min at 37 °C. Fetal bovine serum (30%) in Dulbecco’s modified Eagle’s medium was added to the suspension, and ganglion cells were collected via centrifugation at 400 × *g* for 5 min. The pellet was then resuspended in base medium (Neurobasal medium containing 2% B27, 0.1 mg/ml bovine serum albumin, 0.1 mg/ml transferrin, 1 mM L-glutamine, 5 μg/ml insulin, 1 mM sodium pyruvate, 40 ng/ml triiodothyronine, 40 ng/mL thyroxine, 60 ng/ml progesterone, 16 μg/ml putrescine, 40 ng/ml sodium selenite, 60 μg/ml N-acetyl cysteine, 50 ng/ml brain derived neurotrophic factor (BDNF), 10 ng/ml basic fibroblast growth factor (bFGF), 10 ng/ml ciliary-derived neurotrophic factor (CNTF), 5 mM Forskolin, 100 units/mL penicillin, 100 mg/mL streptomycin). Cells were grown on poly D-lysine-coated petri dishes. Half of the media was changed every third day. The retinal ganglion cell marker anti-Thy1.1 and the neuronal marker anti-neurofilament-L (NF-L, Millipore, Temecula, CA, USA) were used to identify retinal ganglion cells.

### Cell survival assay

Retinal ganglion cells were dispensed in 96-well plates at a density of 5 × 10^4^ cells/100 μl in neurobasal medium. The plate was preincubated overnight in a 37 °C, 5% CO_2_ humidified incubator. The cells were switched to a neurobasal medium containing different concentrations of cobalt chloride (CoCl_2_) for incubation. Cell Counting Kit-8 solution (10 ml) was added to each well of the plate and was incubated at 37 °C for 4 h. The plates were analyzed using an ELISA reader at 450 nm.

### Immunofluorescence

Retinal ganglion cells grown on cover slips were fixed in 4% paraformaldehyde as previously described^7^,^8^,^9^. Briefly, cells were blocked in 5% BSA for 1 h at room temperature, followed by overnight incubation at 4 °C with the following primary antibodies: GPR91 polyclonal antibody (1:200, Novus Biologicals, Littleton, CO, USA), Thy1.1 monoclonal antibody (1:200), c-Fos monoclonal antibody (1:200, Cell Signaling Technology, Boston, MA, USA), C/EBP β monoclonal antibody (1:200, Novus Biologicals, Littleton, CO, USA), and C/EBP δ (1:200, Santa Cruz, CA). The cells were incubated with fluorescein isothiocyanate (FITC)-conjugated anti-rabbit secondary antibodies, phycoerythrin PE-conjugated anti-mouse secondary antibodies or PE-conjugated anti-rabbit secondary antibodies (1:200, Invitrogen, Carlsbad, CA) for 1 h at room temperature. Fluorescence imaging and analyses were performed using a fluorescence microscope (Leica, Wetzlar, Germany).

### Quantitative real-time polymerase chain reaction (qRT-PCR)

Total RNA was extracted from retinas and cells using Trizol reagent (Invitrogen). RNA (2 μg) was used for cDNA synthesis according to the manufacturer’s instructions (Fermentas). The mRNA expression of VEGF, c-Fos, c-Jun, C/EBP α, C/EBP β, C/EBP γ, C/EBPδ, C/EBP ε, C/EBP ζ and β-actin were quantified using real-time quantitative PCR. Each mRNA level was normalized to β-actin mRNA. Specific primers were VEGF (forward: 5′-AAAGCCAGCACATAGGAGAG-3′; reverse: 5′-AGGATTTAAACCGGGATTTC-3′), c-Fos (forward: 5′-CGTCTTCCTTTGTCTTCACCTACC-3′; reverse primer 5′-CTGCCTTCTCTGACTGCTCAC-3′), c-Jun (forward: 5′-GGGCTGTTCATCTGTTTGTCTT-3′; reverse primer 5′-AAGCGGGAGAAGGGACTCT-3′), C/EBP α (forward: 5′-CCAAGAAGTCGGTGGATAAGAA-3′; reverse 5′-GCAGGCGGTCATTGTCACT-3′), C/EBP β (forward: 5′-GGGTTGTTGCTGTTGATGTTTT-3’; reverse 5′-CTCGAAACGGAAAAGGTTCTC-3′), C/EBP γ (forward: 5′-AAGTAAAGAAATCCCATCAGGTCAC-3′; reverse 5′-CCCCAAAGTTCAATCACACTCT-3′), C/EBPδ(forward: 5’-CTGCCATGTATGACGACGAGAG-3′; reverse 5′-GCTTTGTGATTGCTGTTGAAGA-3′), C/EBP ε (forward: 5′-TGCCTATCCCTCACACACATT-3′; reverse 5′-CTCCTCTTTCACCGCCACAG-3′), C/EBP ζ (forward: 5′-ACGAGGAAGACGAGGAGGATAG-3′; reverse 5′-TCCAAAGTAGCCAGCATAAGGTA-3′) and β-actin (forward: 5′-CACCCGCGAGTACAACCTTC-3′; reverse: 5′-CCCATACCCACCATCACACC-3′). Quantitative real-time PCR was completed using SYBR green reagent using a Light Cycler. All reactions were performed in triplicate. Relative changes in gene expression were analyzed using the 2^−ΔΔCT^ method.

### Western blot analysis

Aliquots containing 30 μg of protein were blotted as previously described^7^,^8^,^9^. Briefly, protein samples were analyzed on 10% SDS–PAGE gels and transferred to polyvinylidene difluoride membranes (Millipore, Billerica, MA) in a wet transfer unit (Bio-Rad, Hercules, CA, USA). Membranes were with 5% non-fat dry milk at room temperature for 1 h and incubated overnight at 4 °C with the following primary antibodies: GPR91 polyclonal antibody (1:1000), p-ERK1/2 monoclonal antibody (1:3000, Cell Signaling Technology, Boston, MA, USA), ERK1/2 monoclonal antibody (1:3000, Cell Signaling Technology, Boston, MA, USA), c-Fos monoclonal antibody (1:1000), C/EBP β monoclonal antibody (1:1000), Hypoxia inducible factor-1α (HIF-1α) monoclonal antibody (1:200, abcam, Cambridge, MA, USA), VEGF monoclonal antibody (1:1000, abcam, Cambridge, MA, USA), β-actin monoclonal antibody (1:5000, Sigma Aldrich, Saint Louis, MO, USA). β-Tublin monoclonal antibody (1: 1:1000, abcam, Cambridge, MA, USA) and Histone H3 monoclonal antibody (1:1000, ProteinTech Group, Chicago, IL, USA). Membranes were washed with TBS-Tween 20 and incubated with the appropriate HRP-conjugated secondary antibodies (1:1000, ProteinTech Group, Chicago, IL, USA) for 1 h at room temperature. Bands were visualized using an enhanced ECL detection kit (Bio-Rad, Hercules, CA).

### Enzyme-linked immunosorbent assay (ELISA)

The supernatant medium was collected from non-treated and treated cells for ELISA analysis. VEGF levels in culture supernatants were separately detected using VEGF enzyme-linked immunoassay kits (R&D Systems, Minneapolis, MN, USA) following the manufacturer’s instructions. Each sample was tested in triplicate, and the average results are reported.

### Promoter cloning, site-directed mutagenesis and vector construction

The 2000-bp promoter sequence upstream of the VEGF gene was identified using the UCSC Genome Browser (http://genome.ucsc.edu/), and the potential binding region for C/EBP and AP-1 transcription factors was identified using TFSEARCH (http://www.cbrc.jp). The rat genome was used as a template for PCR amplification of the VEGF promoter fragment (2000 bp), which was subcloned into the pGL3-Basic plasmid (Invitrogen). Mutation of the binding site in the pGL3-VEGF reporter vector construct was performed using site-directed mutagenesis using the QuikChange kit (Stratagene). Mutagenic primers (C/EBP-mut1: forward: 5′-CCTTGAGAGGGAGGGGACAGAGCCA-3′; reverse: 5′-AAGTCCCCTTCACCTCCTCG-3′; C/EBP-mut2: forward: 5′-AGCCCTAGGTCGTCTCCCTCCGGGC-3′; reverse: 5′-GCCCACGTATGCACTGTGGAGT-3′; C/EBP-mut3: forward: 5′-ATCTTAGAGGCGGTGCCTGGTTCGG-3′; reverse: 5′-GCCCCTAGGCCACTACTGCGAAA-3′; AP1-mut1: forward: 5′-GACCGCCGGGCAGCTATGATAGGCCAGA-3’; reverse: 5′-CCTCCCGGATGGCTCTCTTT-3′; AP1-mut2: forward: 5′-ACAGAACCACGCAGGCCTGG-3′; reverse: 5′-AAACCTACCCTAGCATTCAGA-3′) led to a nucleotide change in the entire binding site for the C/EBP and AP1 transcription factors. Mutation was confirmed by nucleotide sequencing.

### Luciferase reporter assays

Reporter assays were performed using the human embryonic kidney cell line HEK293. Cells were seeded on 24-well plates 12 h prior to transfection. Cells were transfected with 40 ng of the pGL3 vector with a VEGF promoter fragment or mutation vector along with 200 ng of a *Renilla* luciferase vector. The plasmid encoding for *Renilla* luciferase vector was used as an internal control for transfection efficiency. Cells were incubated in the transfection media for 6 h, and the media were changed to fresh media. Cells were transfected for 6 h and treated with 400 μM CoCl_2_ for 48 h. Cells were harvested using passive lysis buffer (Dual-Luciferase Reporter Assay System, Promega) and luciferase activity was determined in a single sample luminometer, as described in the manufacturer’s protocol.

### Chromatin immunoprecipitation (ChIP) assay

The cells were cross-linked with 1% formaldehyde, lysed and sonicated to release chromatin. ChIP assays were performed using the EZ ChIP kit per the manufacturer’s instructions (Millipore). The protein-DNA complexes were precipitated using polyclonal antibodies for c-Fos, C/EBP β and C/EBP δ. The extracted DNA was subjected to qRT-PCR with VEGF promoter primers (−115/−129, −893/−907, −1070/−1082, −1304/−1318, −1719/−1731). IgG was used as the negative control to comparedifferences.

### Statistical analysis

All data are presented as the mean ± standard deviation (SD). The data were analyzed using SPSS 16.0 software. The differences between multiple groups were assessed by a one-way ANOVA followed by Student-Newman-Keuls (SNK) comparisons. *P* < 0.05 was considered statistically significant.

## Results

### Effect of GPR91 on VEGF expression in the OIR rat retina

**Figure 1:**
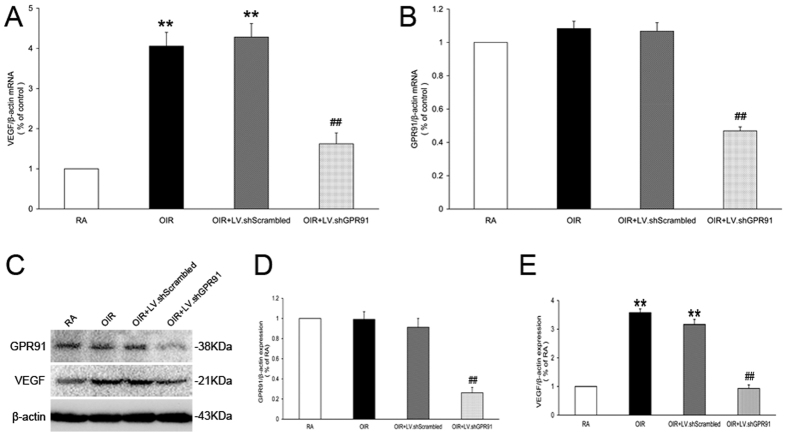
Inhibition of VEGF expression in the retinas of OIR rats following intravitreal injection of LV.shGPR91 particles. (**A**) qRT-PCR analysis of VEGF mRNA in P18 OIR rat retinas and age-matched RA rats. (**B**) qRT-PCR analysis for GPR91 mRNA in P18 OIR rat retinas and age-matched RA rats. (**C**) Western blot analysis of the GPR91 and VEGF proteins in samples from each group. (**D**,**E**) Quantitative analysis of banddensities. Each column denotes the mean ± SD (n = 6). **P < 0.01 versus RA group rats. ^##^*P* < 0.01 versus LV.shScrambled group rats.

We investigated the role of GPR91 in the regulation of VEGF expression in the retinas of OIR rats. At P18, the retinal mRNA and protein levels of VEGF expression increased in OIR rats compared to RA rats (*P* < 0.01, Fig. 1A,C,E). GPR91 knockdown reduced VEGF mRNA in OIR rats by approximately 60% (*P* < 0.01, Fig. 1A) and significantly decreased VEGF protein expression by approximately 70% (*P* < 0.01, Fig. 1C,E). Meanwhile, GPR91 was not significantly different between OIR rats and RA rats (*P* > 0.05, Fig. 1B–D).

### Knockdown of GPR91 attenuated pathological alterations in retinal vessels in OIR rats

**Figure 2:**
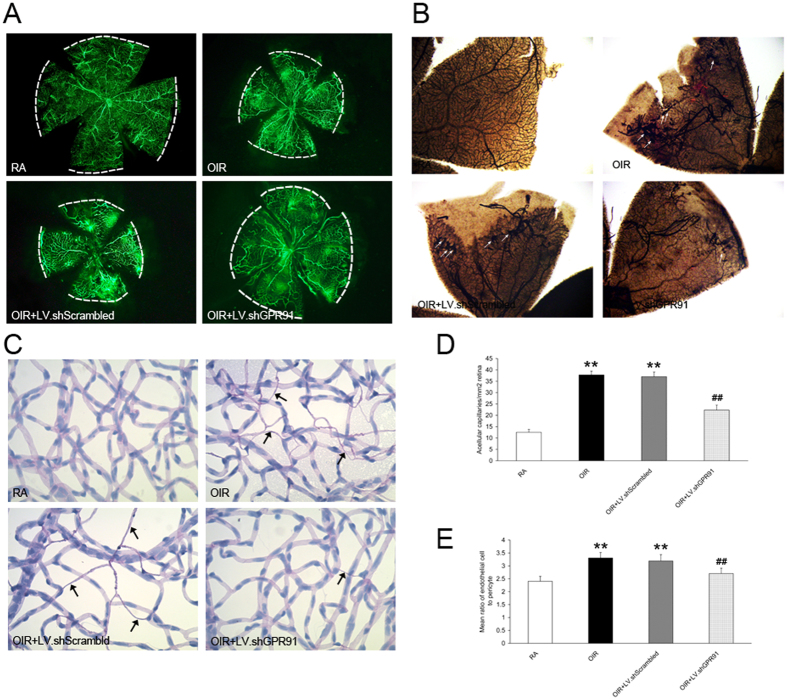
Knockdown of GPR91 expression attenuated pathological alterations in retinal vessels in OIR rats. (**A**) Fluorescein-dextran images of the retinal vessels in P14 rat retinas from each group. *White dashed lines* indicate the boundaries of blood vessels. (**B**) ADPase staining of peripheral retinas in P18 rats from each group. *White arrows* denote abnormal neovascularization tufts. (**C**) Immunohistochemical analyses of trypsin-digested retinal blood vessels in P14 rat retinas from each group. *Black arrows* denote the loss of pericytes. (**D**) Quantitative analysis of the number of acellular capillaries per mm^2^. (**E**) Quantitative analysis of the ratio of endothelial cells to pericytes. Each column denotes the mean ± SD (n = 6). **P < 0.01 versus RA group rats. ^##^*P* < 0.01 versus LV.shScrambled group rats.

Figure 2A,B show a single representative retinal flat mount from each group at P14 and P18, respectively. Retinas were stained with fluorescein-dextran (green) and ADPase (brown). Fluorescein-dextran images at P14 show retinal avascularity in OIR rats (Fig. 2A). The retinal vessels in OIR rats displayed avascular areas in the peripheral retina compared to RA rats, and the central region also exhibited low-grade non-perfusion and tortuosity (Fig. 2A). ADPase staining at higher magnifications revealed that abnormal pre-retinal neovascular tufts arose primarily at the peripheral-most extent of the major veins (Fig. 2B). Immunohistochemical analyses of trypsin-digested retinal blood vessels revealed that the ratio of endothelial cells to pericytes exhibited an upward trend (*P* < 0.01, Fig. 2C,E) and a significant increase in acellular vessels (*P* < 0.01, Fig. 2C,D) in OIR rat retinas. Treatment with GPR91 shRNA attenuated the retinal avascular area and significantly decreased abnormal neovascularization and pericyte loss (*P* < 0.01, Fig. 2A–E), but LV.shScrambled exhibited no such effect (*P* > 0.05, Fig. 2C–E).

### Exposure to CoCl_2_ induces angiogenic factor VEGF release in primary retinal ganglion cells

**Figure 3:**
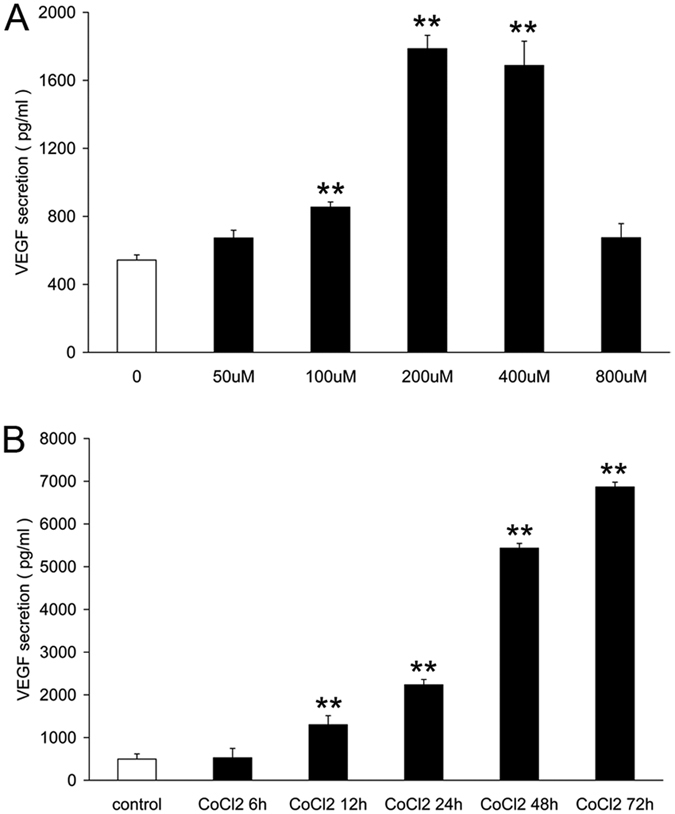
CoCl_2_-induced VEGF release in primary retinal ganglion cells. (**A**) Changes in VEGF secretion (using ELISA) in retinal ganglion cells incubated with different concentrations of CoCl_2_ for 24 h. (**B**) ELISA analysis of VEGF release in retinal ganglion cells incubated with 200 μM CoCl_2_ for 6 h to 72 h. Each column denotes the mean ± SD (n = 3). **P < 0.01 versus untreated control.

The release of VEGF when incubated with control medium or with medium containing different CoCl_2_ concentrations for various time periods was detected to determine whether CoCl_2_ regulated VEGF expression in primary retinal ganglion cells. Figure 3A,B showed that CoCl_2_ exposure notably increased VEGF secretion. Cells incubated with different concentrations of CoCl_2_ for 24 h exhibited a dose-dependent increase in VEGF release, and a time-dependent trend was also observed when the cells were incubated with 200 μM CoCl_2_. VEGF secretion was upregulated at least 4-fold in primary retinal ganglion cells after incubation with 200 μM CoCl_2_ for 24 h (Fig. 3A,B).

### CoCl_2_ affects the survival rate of primary retinal ganglion cells

**Figure 4:**
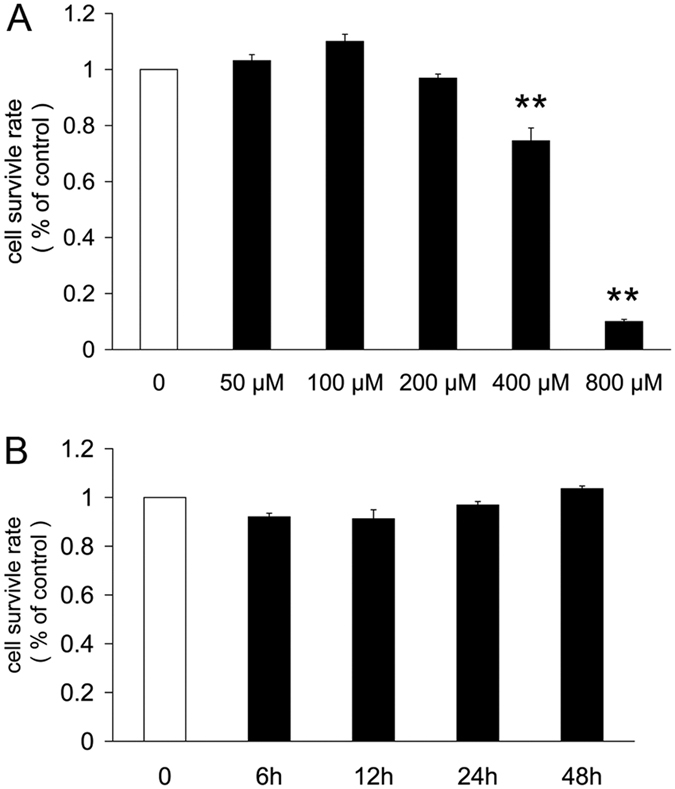
Effect of CoCl_2_ on the survival rate of retinal ganglion cells. (**A**) The survival rate of retinal ganglion cells incubated with different concentrations of CoCl_2_ for 24 h. (**B**) CCK8 analysis of the survival rate in retinal ganglion cells incubated with 200 μM CoCl_2_ for 6 h to 72 h. Each column denotes the mean ± SD (n = 3). **P < 0.01 versus untreated control.

We used CCK-8 assays to observe the survival of primary retinal ganglion cells after incubation with control medium or medium containing CoCl_2_. Retinal ganglion cells exhibited reduced survival rates for 24 h at 400 μM and 800 μM conditions compared to control medium or other doses of CoCl_2_ medium (*P* < 0.01). However, there were no obvious changes between the groups incubated with controls or 200 μM CoCl_2_ medium for different time periods (Fig. 4A,B).

### GPR91 in primary retinal ganglion cells and transient transduction of ganglion cells with GPR91 siRNA

**Figure 5:**
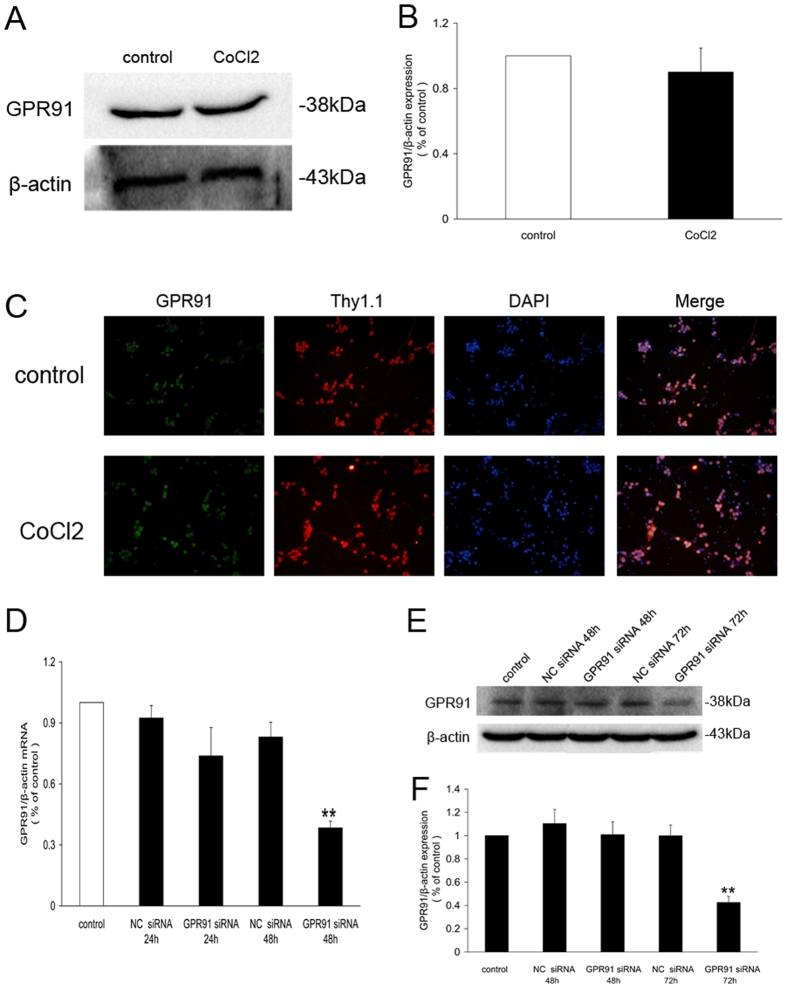
Distribution of GPR91 in the retinal ganglion cells. (**A**) Western blot analysis of GPR91 protein expression in retinal ganglion cells treated with CoCl_2_ for 24 h. (**B**) Quantitative analysis of band density. Each column denotes the mean ± SD (n = 3). (**C**) Immunofluorescence showing GPR91 expression in the cytoplasm of retinal ganglion cells. Green fluorescence shows the distribution of the GPR91 protein. Red fluorescence shows the distribution of the retinal ganglion cell marker Thy1.1 protein. Blue fluorescence shows nuclei stained with 4,6-diamidino-2-phenylindole (DAPI). The right column shows the merged pictures. (**D**) Changes in mRNA levels (as determined using qRT-PCR) of GPR91 in samples from each group. (**E**) Western blot analysis of the GPR91 protein in samples from each group. (**F**) Quantitative analysis of the band density of GPR91/β-actin. Each column denotes the mean ± SD (n = 3). **P < 0.01 versus the NC 72 h group.

We investigated the expression and location of the GPR91 receptor to determine whether CoCl_2_ modulates GPR91 expression. Western blot experiments revealed no significant differences in GPR91 protein levels in primary retinal ganglion cells incubated with 200 μM CoCl_2_ for 24 h (Fig. 5A,B). Immunofluorescence revealed that GPR91 was predominantly localized to the cell bodies of retinal ganglion cells incubated with or without CoCl_2_ (Fig. 5C).

Next, we studied GPR91 expression using an siRNA. The levels of GPR91 mRNA and protein at 48 h and 72 h, respectively, were obviously reduced in cells transduced with GPR91 siRNA compared to cells transduced with NC siRNA (Fig. 5D–F).

### GPR91 modulates the CoCl_2_-induced increase in ERK1/2 signaling and C/EBP, AP-1 activity in primary retinal ganglion cells

**Figure 6:**
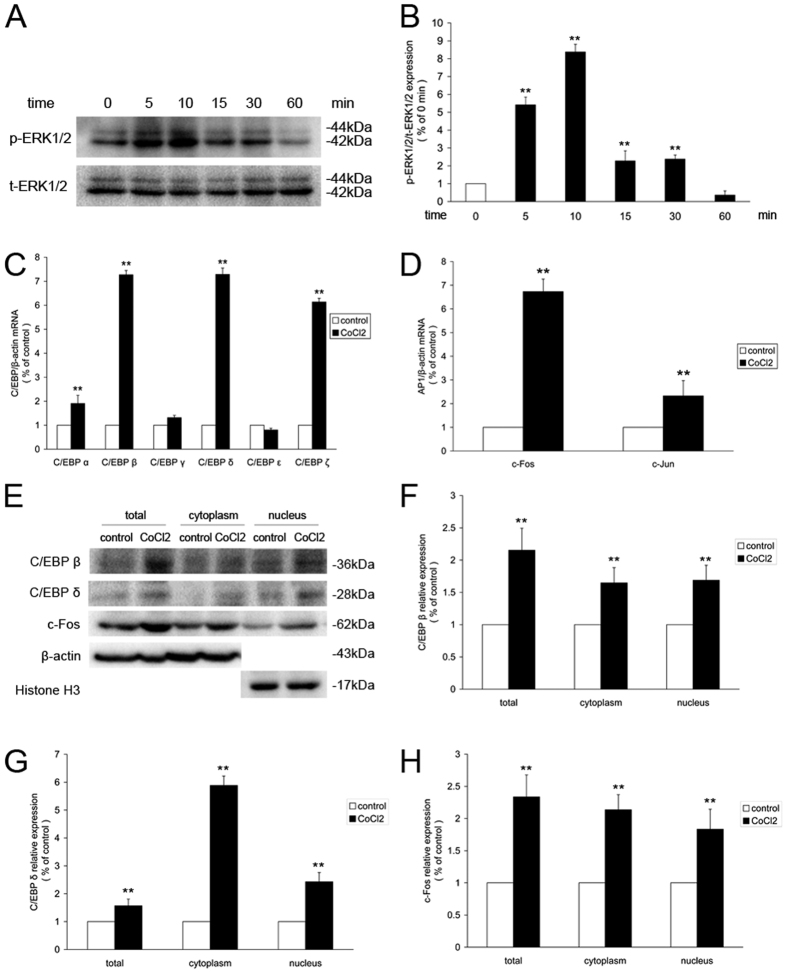
CoCl_2_-induced activation of ERK1/2 signaling, C/EBP and AP-1 in retinal ganglion cells. (**A**) Western blot analysis of ERK1/2 phosphorylation in retinal ganglion cells treated with 200 μM CoCl_2_ for different periods of time. (**B**) Quantitative analysis of the band density of p-ERK1/2/t-ERK1/2. (**C**) qRT-PCR analysis of C/EBP subtypes in retinal ganglion cells treated with 200 μM CoCl_2_ for 24 h. (**D**) qRT-PCR analysis of AP-1 subtypes in retinal ganglion cells treated with 200 μM CoCl_2_ for 24 h. (**E**) Changes in C/EBP β, C/EBP δ and c-Fos protein levels in CoCl_2_-induced retinal ganglion cells treated for 24 h. (**F**–**H**) Quantitative analysis of the band density. Each column denotes the mean ± SD (n = 3). **P < 0.01 versus control. ^##^*P* < 0.01 versus the NC siRNA group. ^ψψ^*P* < 0.01 versus the CoCl_2_ group.

**Figure 7:**
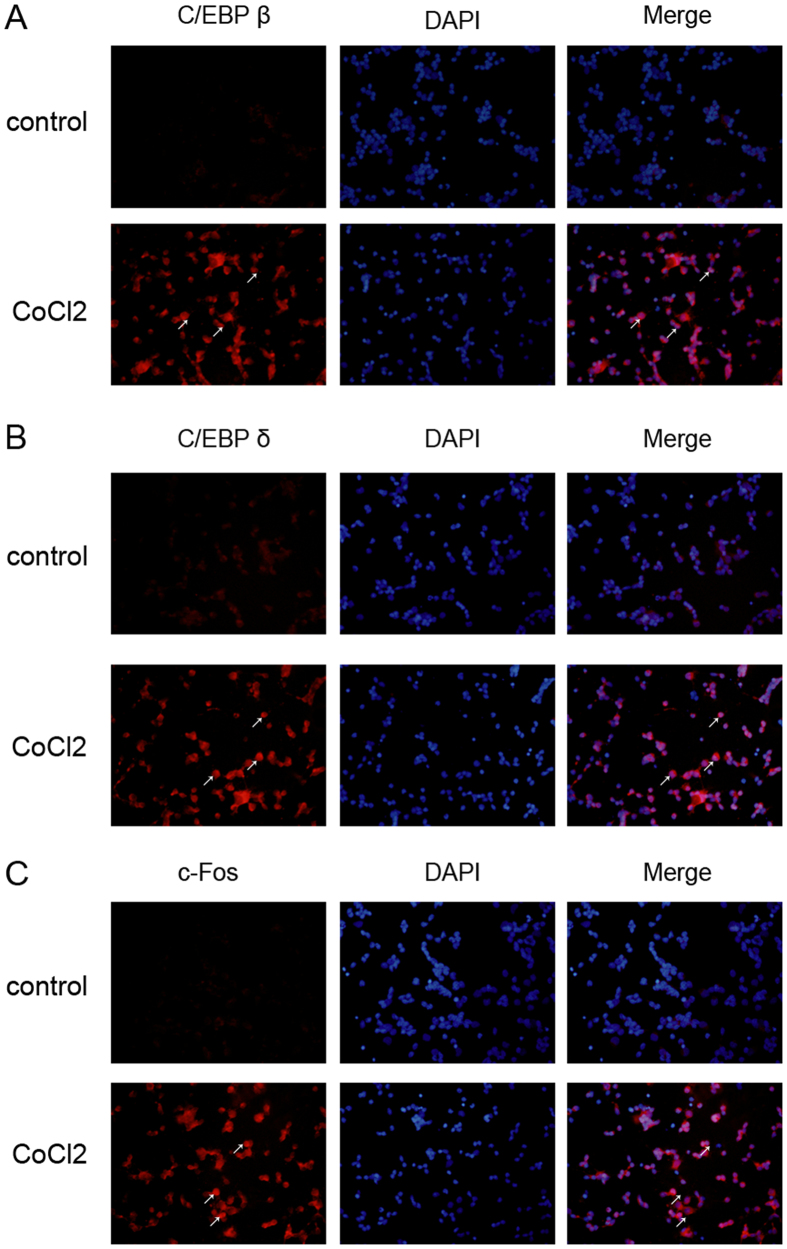
Immunofluorescence images of C/EBP and AP-1 activation in retinal ganglion cells. (**A**) Immunofluorescence shows increased C/EBP β increased in the cytoplasm and nucleus of retinal ganglion cells after incubation with 200 μM CoCl_2_ for 24 h. (**B**) The expression of C/EBP δ was upregulated in the cytoplasm and nucleus of the retinal ganglion cells after incubation with 200 μM CoCl_2_ for 24 h. (**C**) Enhanced c-Fos protein located in the cytoplasm and nucleus of retinal ganglion cells after incubation with 200 μM CoCl_2_ for 24 h. The left column shows the distribution of C/EBP β, C/EBP δ or c-Fos proteins in red fluorescence. The central column shows nuclei stained with DAPI (blue fluorescence). The right column shows the merged pictures.

We then identified the presence and importance of ERK1/2 signaling and C/EBP and AP-1 transcription factor in CoCl_2_-treated primary retinal ganglion cells. Western blot demonstrated that ERK1/2 phosphorylation peaked in cells incubated with 200 μM CoCl_2_ for 10 min (Fig. 6A,B). qRT-PCR revealed various C/EBP isoforms (C/EBP α, C/EBP β, C/EBP δ, C/EBP ζ) and AP-1 isoforms. c-Fos and c-Jun were upregulated after 200 μM CoCl_2_ for 24 h (Fig. 6C,D). CoCl_2_ induced a > 6-fold increase in C/EBP β, C/EBP δ, C/EBP ζ and c-Fos mRNA levels in retinal ganglion cells. Western blot found that C/EBP β, C/EBP δ and c-Fos expression increased in the cytoplasm and nuclei of retinal ganglion cells (Fig. 6E–H). Immunofluorescence demonstrated that C/EBP β, C/EBP δ and c-Fos became notably more visible in the cytoplasm and nucleus after incubation with 200 μM CoCl_2_ for 24 h and exhibited nuclear translocation (Fig. 7A,B).

### Mutation of the C/EBP and AP1 binding sites decreases VEGF promoter activity in HER293 cells

**Figure 8:**
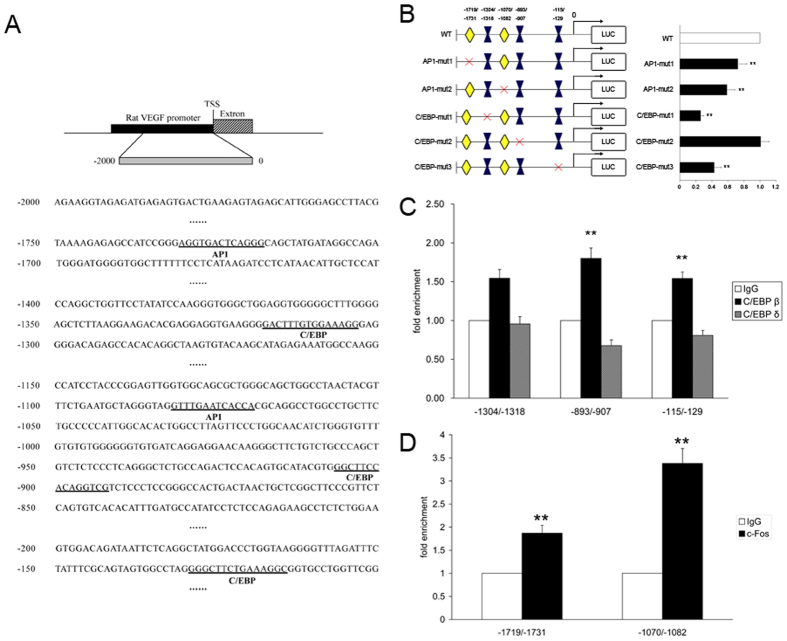
Identification of functional binding sites for C/EBP and AP-1. (**A**) The potential binding sites for C/EBP and AP-1 transcription factors in the 2000-bp promoter sequence upstream of the VEGF gene. The numbers indicate the base location relative to the transcription start site. TSS: transcription start site. (**B**) Luciferase reports showing changes in VEGF promoter activity after mutations of the binding region using HEK293 incubated with 400 μM CoCl_2_ for 24 h. (**C**) A ChIP assay (using qRT-PCR) for C/EBP β and C/EBP δ using retinal ganglion cells incubated with 200 μM CoCl_2_ for 24 h. (**D**) A ChIP assay (using qRT-PCR) for c-Fos using retinal ganglion cells incubated with 200 μM CoCl_2_ for 24 h. Each column denotes the mean ± SD (n = 3). **P < 0.01 versus IgG.

We used a gene mutagenesis technique to mutate C/EBP and AP1 binding site regions as follows: C/EBP-mut1 (−1304/−1318): gactttgtggaaagg to gactt**CCTT**ga**G**agg, C/EBP-mut2 (−893/−907): ggcttccacaggtcg to ggc**AG**c**CCT**aggtcg, C/EBP-mut3 (−115/−129): gggcttctgaaaggc to gggc**A**tct**TAGA**ggc, AP1-mut1(−1719/−1731): aggtgactcaggg to agg**GACCG**c**C**ggg, and AP1-mut2 (−1070/−1082): gtttgaatcacca to gttt**ACAGAA**cca (Fig. 8A). HER293 cells were incubated with 400 μM CoCl_2_, and luciferase activity of mutations in the C/EBP-mut1 and C/EBP-mut3 sites was reduced by at least 70% and 50%, respectively. Mutations in the AP1-mut1 and AP1-mut2 site resulted in a 27% and 40% decrease in luciferase activity, respectively (Fig. 8B). However, the binding site of C/EBP-mut2 exhibitited no change in luciferase activity.

### C/EBP β and c-Fos binds the VEGF promoter in the nucleus

We performed chromatin immunoprecipitation using an anti-C/EBP β, anti-C/EBP δ and anti-AP1 antibody after primary retinal ganglion cells were incubated with 200 μM CoCl_2_ to observe combinations of C/EBP β and C/EBP δ with VEGF promoter in the −115/−129, −893/−907 and −1304/−1318 regions and c-Fos in the −1070/−1082 and −1719/−1731 regions. The relative enrichment of the −115/−129, −893/−907 and −1304/−1318 regions was efficiently increased when a C/EBP β antibody was used, and the c-Fos antibody also induced the same trend in the −1070/−1082 and −1719/−1731 regions (Fig. 8C). In contrast, we did not detect any differences in the relative enrichment of C/EBP δ antibodies (Fig. 8D).

### C/EBP β, c-Fos and HIF-1α regulate the GPR91-mediated expression of VEGF in primary retinal ganglion cells

**Figure 9:**
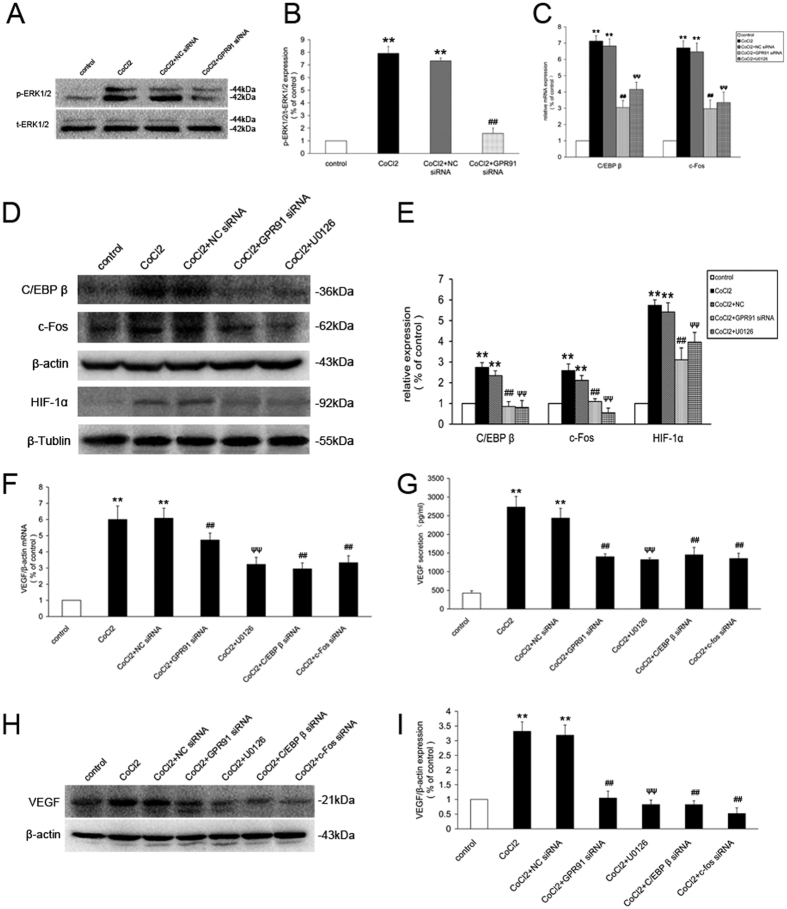
GPR91 modulated the CoCl_2_-induced increase in ERK1/2 signaling, C/EBP β, c-Fos, HIF-1α activity and VEGF expression in retinal ganglion cells. (**A**) Changes in ERK1/2 phosphorylation (using western blot) in retinal ganglion cells transduced with LV.shScrambled or LV. shGPR91. (**B**) Quantitative analysis of band density. Each column denotes the mean ± SD (n = 3). (**C**) qRT-PCR analysis for C/EBP β and c-Fos mRNA in retinal ganglion cells from each group. (**D**) Western blot analysis of C/EBP β, c-Fos and HIF-1α protein expression in retinal ganglion cells treated with CoCl_2_ for 24 h. The cells were transduced with GPR91 siRNA or pre-treated with U0126 (ERK1/2 inhibitor). (**E**) Quantitative analysis of band density. (**F**) Changes in mRNA levels of HIF-1αVEGF in samples from each group. (**G**) ELISA analysis of VEGF secretion in samples from each group. (**H**) Western blot analysis of VEGF secretion in samples from each group. (**I**) Quantitative analysis of band density. Each column denotes the mean ± SD (n = 3). Each column denotes the mean ± SD (n = 3). **P < 0.01 versus control. ^##^*P* < 0.01 versus NC siRNA group. ^ψψ^*P* < 0.01 versus CoCl_2_ group.

We evaluated ERK1/2 signaling and C/EBP β, c-Fos and HIF-1α activation in cells transduced with NC siRNA or GPR91 siRNA incubated with or without 200 μM CoCl_2_ to confirm the signaling mechanism of GPR91. GPR91 siRNA significantly inhibited the increase in ERK1/2 phosphorylation levels, C/EBP β, c-Fos and HIF-1α expression (Fig. 9A–E). The mRNA and protein levels of C/EBP β and c-Fos and HIF-1α expression were downregulated in ERK1/2 inhibitor U0126-treated cells compared to the cells incubated with CoCl_2_ (Fig. 9C–E).

We investigated the relationship between GPR91 and the CoCl_2_-stimulated increase in VEGF.VEGF expression and secretion by these cells was significantly downregulated by transduction with GPR91 siRNA, C/EBP β siRNA or c-Fos siRNA, and pretreatment with 10 μM U0126 significantly dowmregulated VEGF expression and secretion by these cells (*P* < 0.01, Fig. 9F–I).

### GPR91-mediated ERK1/2/ C/EBP β (c-Fos, HIF-1α) /VEGF activity in the OIR rat retina

**Figure 10:**
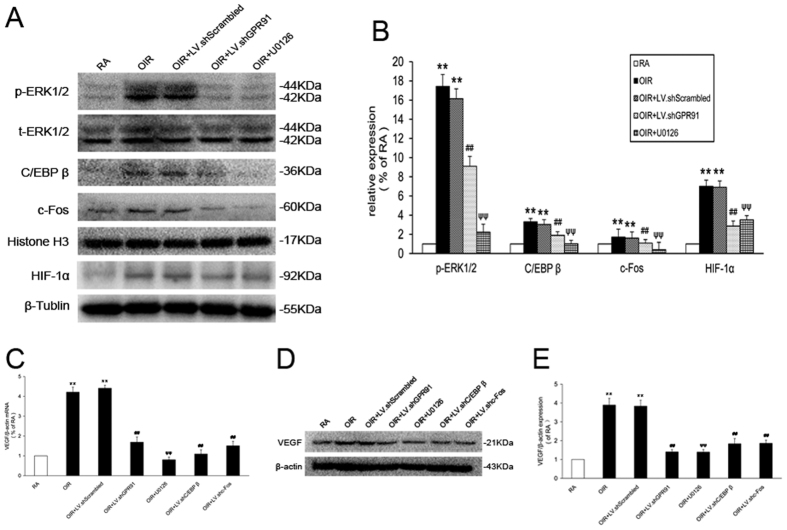
GPR91 modulated the increase in VEGF via the ERK1/2/ C/EBP β (c-Fos, HIF-1α) pathway in OIR rats. (**A**) Western blot analysis of ERK1/2 signaling, C/EBP β, c-Fos and HIF-1α activation in samples from each group. (**B**) Quantitative analysis of band density. (**C**) qRT-PCR analysis for VEGF mRNA in retinal ganglion cells from each group. (**D**) Western blot analysis of VEGF expression in samples from each group. (**E**) Quantitative analysis of the band density. Each column denotes the mean ± SD (n = 3). **P < 0.01 versus RA rats. ^##^*P* < 0.01 versus LV.shScrambled group rats. ^ψψ^ *P* < 0.01 versus OIR group.

The retinas of P18 OIR rats also exhibited a significant increase in p-ERK1/2, C/EBP β, c-Fos and HIF-1α levels compared to the RA rats (Fig. 10). However, intravitreal injections of LV.shGPR91 or 0.1 mM U0126 downregulated these increases in p-ERK1/2, C/EBP β, c-Fos and HIF-1α (Fig. 10). LV.shC/EBP β or LV.shc-Fos significantly blocked C/EBP β and c-Fos activation and VEGF expression (*P* < 0.01, Fig. 10).

## Discussion

Our previous research demonstrated that succinate were increased in the retina of streptozotocin-induced diabetic rats, and succinate regulated VEGF expression via activation and binding to its special receptor in the ganglion neural cells (GCL)^7^. Succinate is an intermediate of the Krebs cycle that is associated with metabolic demands under physiological conditions. However, pathological situations, such as hyperglycemia, ischemia and hypoxia disrupt the flow of substrates in this cycle, which further increases succinate levels^4^,^6^. Our current results demonstrate that lentivirus-delivered GPR91 shRNA attenuated the hypoxia-induced pathological alterations in retinal vessels in the retinal avascular area, abnormal neovascularization and pericyte loss, which is similar to Sapieha’s findings in their OIR rat model^4^. These results suggest that hypoxia-induced GPR91 activation may affect original retinal vessels and accelerate the pathological course of ischemic retinopathy.

VEGF is a pivotal regulator in various retinopathies, but the regulation of VEGF expression is complicated: pre-transcription, transcription initiation, post-transcription processing, translation and modification after translation. The level of transcription initiation is one of the most common links in the regulation of gene expression. Transcription initiation is a more complex process in eukaryotic cells. Eukaryotic RNA polymerase does not directly recognize the core promoter sequences. A collection of transcription factors are attached to the promoter sequences via binding to the RNA polymerase, which form a transcription initiation complex that regulates the initiation of transcription^25^. Transcription factors control the expression of a given gene by serving as integration centers of different signaling cascades^26^. Many transcription factors regulate VEGF transcription and expression^27^,^28^,^29^. Daft *et al*.^27^ found that the inhibition of CaMKII and VEGFR decreased HIF-1α and AP-1 binding to the VEGF promoter, which is likely responsible for the observed decreases in VEGF transcription. One report demonstrated that hyperglycemia significantly increased the binding of the transcription factor Sp1 to the VEGF-A promoter^28^. Du *et al*.^29^ used site-directed mutagenesis, electrophoretic mobility shift assay (EMSA) and ChIP technologies to determine the nuclear factor (NF)-κB binding site on the VEGF-C promoter. Our *in vivo* experimental results demonstrated that GPR91 upregulated VEGF expression in the GCL of the rat retina via ERK1/2/COX-2/PGE_2_ signaling pathway activation^7^, but the action of GPR91 on the transcription initiation process of the VEGF gene in hypoxic retinopathy is not known. Therefore, our current research primarily focused on the transcription level to examin the regulatory mechanisms of GPR91-modulated VEGF expression and secretion.

Our previous work used the TFSEARCH website to predict the transcription factors of the sequence 2000 bp upstream of the VEGF-A promoter and found that C/EBP and AP-1 are eligible transcription factors with scores >90 and that belong to the rat sources. Our study demonstrated at differential expression of C/EBP and AP1 subtypes under CoCl_2_ stimulation, and C/EBP β, C/EBP δ, C/EBP ζ and c-Fos were significantly increased, which suggeststhat these four subtypes may be involved in the regulation of VEGF transcription. We also observed three putative C/EBP binding sites (−115/−129, −893/−907 and −1304/−1318) and two putative AP-1 binding sites (−1070/−1082 and −1719/1731) inthe 2000-bp WT-VEGF gene promoter. The promoter sequence of the rat VEGF gene was selectively mutated at five predicted C/EBP and AP-1 putative sites in the present study. Luciferase reporter analyses revealed that the activation of two of the C/EBP binding sites (−115/−129 and −1304/−1318) and one of the AP-1 binding sites (−1070/−1082) were notably downregulated compared to the WT-VEGF gene promoter in HER293 cells. ChIP analyses revealed a significant enrichment of C/EBP β in −115/−129, −893/−907 and −1304/−1318 regions, and c-Fos also exhibited changes in the −1070/−1082 and −1719/1731 regions under CoCl_2_ conditions in retinal ganglion cells. C/EBP δ was not affected. The upregulation of C/EBP δ under hypoxic conditions was also reported in a study of lymphatic endothelial cells, and these results are similar to ours^30^. However, Min Y *et al*.^15^ found that C/EBP δ was associated with the transcriptional regulation of VEGF-C. Our study mainly focused on the role of VEGF-A, which may explain the absence of change in C/EBP δ in our CHIP analysis.

Other groups also identified that C/EBP β and c-Fos were highly sensitive to hypoxic conditions^31^,^32^,^33^,^34^. Our research demonstrated that C/EBP β and c-Fos were enhanced to some extent in OIR rats and CoCl_2_-induced primary retinal ganglion cells. Immunofluorescence also showed that activated C/EBP β and c-Fos were transferred from the cytoplasm to the nucleus where these protein participated in transcription regulation. The upregulation of C/EBP β and c-Fos activity and function in hypoxic states is potentially problematic for the retina. However, Herr KB *et al*.^35^ reported that chronic intermittent hypoxia reduced the baseline activity of c-Fos in medullary catecholaminergic neurons. C/EBP β are regulated at several levels, including gene transcription, translation, protein-protein interactions, phosphorylation-mediated changes in DNA binding activity, activation potential and nuclear localization^12^. C/EBP βalso responds to various extra- and intracellular signals to modulate the expression of many genes, including genes involved in proliferation, differentiation, the inflammatory response, metabolism and apoptosis^36^. c-Fos is generally among the first gene expressed and is therefore referred to as an immediate early gene, which is rapidly and transiently induced^37^. A variety of stimuli, including serum, growth factors, tumor promoters, cytokines, hypoxia, and UV radiation, induce c-Fos expression^17^. C/EBP β and c-Fos are potent inducers of neovascularization in various diseases^38^,^39^. A recent study showed that C/EBP β bound to the promoter region of VEGF-C to induce VEGF-C expression in IL-6-exposed lymphatic endothelial cells^16^. Lee *et al*.^40^ reported that inactivation of C/EBP β suppressed VEGF expression. Catar R *et al*.^41^ demonstrated that c-Fos inhibition small interfering RNA decreased VEGF promoter activity and downregulated its expression and release. Therefore, we hypothesized that the role of C/EBP β- and c-Fos-modulated angiogenesis is at least partially accomplish by regulation of the transcription initiation of VEGF.

To further confirm the relationship between transcription factors and VEGF, we used siRNA or lentiviral vehicle to reduce the combination of C/EBP β or c-Fos. Our experiments demonstrated that the RNA and protein levels of VEGF expression and secretion were significantly blocked in OIR rats and CoCl_2_-induced primary retinal ganglion cells. These results indicated that C/EBP β and c-Fos could be the main subtypes in the regulation of the VEGF transcription process and affected changes in the retinal vessels in hypoxia-induced retinopathy. Whether GPR91 is involved in C/EBP β, and c-Fos expression is poorly understood. In addition, hypoxia-triggered events have classically been considered to depend on pathways involving HIF-1α, which is known transcription factor of VEGF^42^,^43^. Widely research had identified the interaction between HIF-1α and VEGF and the combinations site of HIF-1α in VEGF promoter^44^,^45^,^46^. Recent study exhibited that succinate inhibited HIF prolyl-hydroxylase to stabilize HIF-1α^47^. It indicated that succinate-GPR91 signaling may played significant role in regulating HIF-1α expression under hypoxia conditions. We reduced GPR91 expression and activation and found that these transcription factors were downregulated. These above results illustrated that the hypoxia-induced C/EBP β,c-Fos and HIF-1α transcription factors are important regulators of GPR91-mediated VEGF gene transcription in the nucleus. However, whether there is mutual regulatory mechanism between these transcription factors remains to be further explored.

Multiple studies demonstrated that the activity of C/EBP β, c-Fos and HIF-1αis also regulated by posttranslational modification caused by phosphorylation by different kinases, such as MAPK, cdc2, PKA and PKC^16^,^17^,^48^,^49^,^50^. Previous studies targeted the effects of ERK1/2 pathways on GPR91-dependent VEGF expression in the retinas of streptozotocin-induced diabetic rats^7^. Our current investigation also established that p-ERK1/2 and VEGF were upregulated in parallel under hypoxic conditions in primary retinal ganglion cells, and this phenomenon was entirely GPR91-specific. We also verified the effect of the activation of ERK1/2 signaling on C/EBP β, c-Fos and HIF-1α regulation in hypoxia-induced retinopathy.

ERK1/2 is one of the classical MAPK signaling pathways thatregulates specific transcription factors by enhancing their stability and transcriptional activity and controlling cellular growth, differentiation, and survival^51^. The ERK1/2 pathway is involved in the expression of multiple downstream biological signals, includingregulation of the expression of C/EBP β, c-Fos and HIF-1α. Lin *et al*.^52^ found that thrombin-induced C/EBP β activation was blocked by the ERK inhibitor PD98059, and ERK signaling promoted the formation of a C/EBP β-specific DNA complex. A breast cancer study reported that upregulation of the ERK signaling pathway initiated further C/EBP β activation^53^. One recent report found that the inhibition of ERK1/2 signaling blocked c-Fos activation^54^. Guo C *et al*.^50^ found that desferrioxamine-mediated up-regulation of HIF-1α occurred via the activation of the ERK signaling pathway in SH-SY5Y cells. Administration of the ERK1/2 inhibitor U0126 in the presence of hypoxia abolished C/EBP β, c-Fos and HIF-1α production, which provides further evidence of the role of C/EBP β, c-Fos and HIF-1α activation by ERK1/2 phosphorylation, which is consistent with the results of previous research. These data provide further confirm that ERK1/2-derived C/EBP β, c-Fos and HIF-1α activation increases in response to low oxygen conditions and GPR91-dependent VEGF release.

In conclusion, activation of the GPR91-ERK1/2-C/EBP β (c-Fos, HIF-1α) signaling pathway plays a tonic role in the regulation of VEGF transcription in rat retinal ganglion cells. VEGF expression is dynamically and strictly regulated by the positive signaling pathways of ERK1/2, C/EBP β, c-Fos and HIF-1α. This intricate and orchestral regulation may aid in maintaining the transient function of VEGF in retinal vascular damage. Our study of the regulation of hypoxia-induced VEGF expression by GPR91 in retinal ganglion cells suggests that vascular lesions and nerve damage do not exist in isolation in ischemic retinopathy, but these processes overlap and maintain intricate contact with each other. The interaction of multiple pathways regulates VEGF secretion in hypoxic retinopathy. Therefore, the inhibition of their function will likely interfere with the function of numerous signaling molecules that are inhibited during the progression of hypoxic retinopathy, which occurs in DR, ROP and occlusive diseases of the retinal vessels.Thus, the knowledge acquired from the present study provides helpful clues for the translation of this new knowledge into clinical practice.

## Additional Information

**How to cite this article:** Li, T. *et al*. Transcription factors regulate GPR91-mediated Eexpression of VEGF in hypoxia-induced retinopathy. *Sci. Rep.* **7**, 45807; doi: 10.1038/srep45807 (2017).

**Publisher's note:** Springer Nature remains neutral with regard to jurisdictional claims in published maps and institutional affiliations.
